# Effect of Disinfectants on Mechanical Properties of Orthodontic Acrylics

**DOI:** 10.1155/2019/1096208

**Published:** 2019-04-24

**Authors:** Tobias Bensel, Jens J. Bock, Anne Kebernik, Christin Arnold, Sonia Mansour, Arne F. Boeckler

**Affiliations:** ^1^Department of Prosthodontics, Martin-Luther-University Halle-Wittenberg, Magdeburger straße 16, 06112 Halle, Germany; ^2^Private Orthodontic Practice, Im Schloßgarten 1, 36037 Fulda, Germany

## Abstract

**Objective:**

Infection control protocols in dentistry dictate that orthodontic acrylics have to be disinfected. No specific products for orthodontic acrylics are available. The objective of this study was to investigate the influence of chemical disinfectants on mechanical properties of orthodontic acrylics.

**Materials and Methods:**

260 test specimens of two cold-curing orthodontic acrylics were manufactured. Three chemical disinfecting agents were tested: Impresept, D050 Instru-Gen, and Stammopur DR. Test specimens were stored in distilled water and divided into test groups. E-Modulus, flexural strength, macro hardness, micro hardness, average roughness, and colour change were measured.

**Results:**

Disinfection agents showed no significant influence on E-modulus. Values ranged from 1783.80 ± 163.80 MPa (Forestacryl colourless) to 2474.00 ± 135.00 MPa (Orthocryl green) after storage in distilled water. Disinfection agents performed no significant influence on flexural strength. Values ranged from 18.64±1.59 N/mm^2^ (Forestacryl colourless) to 25.64 ± 1.43 N/mm^2^ (Orthocryl green) after storage in distilled water. Orthocryl colourless showed a reduction of the macro hardness after disinfection (Stammopur DR (p≤0.001), D050 Instru-Gen (p≤0.037)). Disinfection of Orthocryl green with D050 Instru-Gen (p<0.001) and Forestacryl colourless with Impresept (p≤0.001) led to a reduction of macro hardness. Micro hardness of Orthocryl colourless altered significantly after disinfection with D050 Instru-Gen (p≤0.001). Micro hardness of Forestacryl colourless increased (Impresept (p≤0.039)) and decreased (Stammopur DR (p≤0.006) Instru-Gen (p≤0.001)) after disinfection. Average roughness did not change significantly (Orthocryl colourless). Forestacryl colourless performed a significant change after disinfection with Stammopur DR (p≤0.05). This is also true for the disinfection of Orthocryl green and Forestacryl pink with Instru-Gen (p≤0.05). Disinfection performed no significant influence on colour change. ΔE-values were in a range of 1 to 2.

**Conclusions:**

Some orthodontic acrylics disinfection caused significant changes of determined parameters. Changes were specific for the applied disinfectant and tested orthodontic acrylic. Further studies should verify the impact of long-term disinfection intervals. Thus, from manufacturers of orthodontic acrylics recommendations for appropriate disinfectants would be desirable.

## 1. Introduction

The oral cavity is a reservoir for opportunistic and pathogenic microorganisms. During routine orthodontic and dental practice, there is a high risk of cross-contamination and infection that may even cause systemic infections [1]. Transfer of pathogens from patients to members of the orthodontic team occurs and vice versa. Transfer of pathogens may also take place from patient to patient without intervention of the orthodontic team. These transmission routes consist of contaminated surfaces, instruments, orthodontic equipment, or dentures. In general, the major vectors of cross-transmission are the patient's saliva and blood [2, 3]. Overall, there might be a high risk of infection for all participants of orthodontic treatment. This includes patients, providers, orthodontic assistants, and laboratory technicians [4].

Omitting a proper disinfection of orthodontic equipment might increase the risk for transmission of communicable diseases and facultative pathogenic microorganisms. Dental technicians show a higher rate of hepatitis-B-infestation compared to the average population [5, 6]. Therefore, there is a need to reduce the possible transmission routes of pathogens in the orthodontic and dental setting.

The need to disinfect orthodontic equipment before and after contact with patients is theoretically an obligation [6, 7]. Orthodontic equipment contacts mucous membranes and should be cleaned of all microorganisms before handling or adjusting them. These items require high-level disinfection using chemical disinfectants. The intention of the chemical disinfecting process is the inactivation of pathogens without affecting the material structure of dentures and orthodontic acrylics [8]. The ultimate aim of the process of disinfection of orthodontic equipment should be the elimination of enough pathogens to prevent transmission of infection [6, 9, 10].

There are no agents produced specifically for disinfection of orthodontic equipment. However, disinfecting agents originally produced for disinfection of dental impressions are used commonly. In the majority of cases, aldehydes are the utilized agents for disinfecting orthodontic equipment and acrylic dentures. The disinfecting effect of dentures using aldehydes is adequate, although the material safety data sheets give no direct information regarding disinfection of orthodontic equipment and acrylic dentures [11].

Thus, the purpose of this* in vitro* study was to determine the effect of various chemical disinfection agents on the material properties of different orthodontic acrylics. Four different orthodontic acrylics and three disinfecting agents were used during this investigation. The effects of the chemical disinfecting process on modulus of elasticity, flexural strength, macro hardness, micro hardness, surface roughness, and colour changes of the orthodontic acrylics were determined.

The hypothesis of this study is that the investigated chemical disinfecting agents have a significant influence under simulated practice conditions (single use disinfection) on the modulus of elasticity, the flexural strength, the macro hardness, the micro hardness, the average roughness, and colour change of different orthodontic acrylics.

## 2. Material and Methods

### 2.1. Test Specimen and Disinfecting Agents

**Figure 1:**
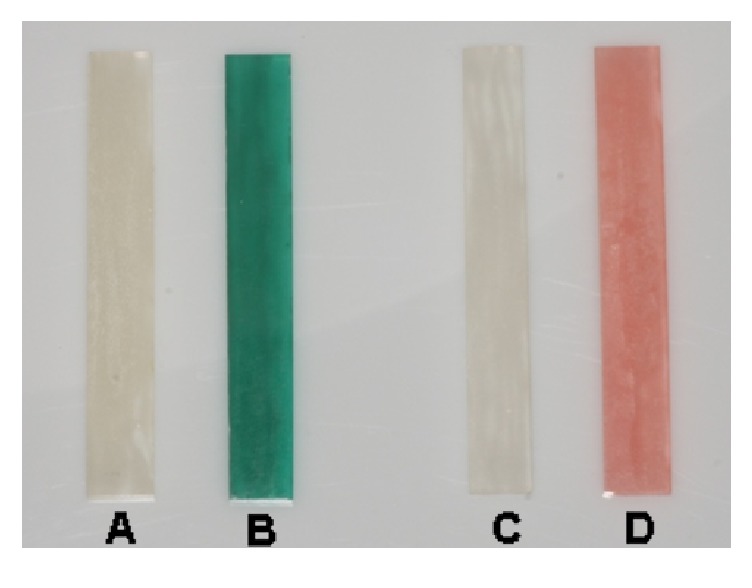
Cold-curing orthodontic acrylics after polymerisation. (A) Orthocryl colourless; (B) Orthocryl green; (C) Forestacryl colourless; and (D) Forestacryl pink.

For this investigation, 260 specimens consisting of two different orthodontic acrylics were fabricated. The cold-curing polymer Orthocryl in the colours green and colourless (Dentaurum, Ispringen, Germany) and the cold-curing polymer Forestacryl in the colours pink and colourless (ForestaDent, Pforzheim, Germany) were used. Test specimens were designed prismatically according to DIN EU ISO Norm 3167:2003 by using spreading technology and were constructed with dimensions of 80x10x4 mm. All test specimen surfaces were ground and polished, using granulation sizes of 220, 320, 800, 1200, and 2400 (RotoPol-35, Struers GmbH, Willich, Germany) (Figure 1).

The test specimens were distributed into test groups. Ensuring standardized water saturation and for appearing the maximum water sorption, 198 test specimens were inserted at 22°C in distilled water for 24 hours and finally incubated at 22°C (WTC Binder, Tuttlingen, Germany) aerobically for 24 hours. Finally, the influence of the disinfecting agents, consisting of Impresept (3M Espe, 3M Company, St. Paul, Minnesota, U.S.A.), Stammopur DR (Dr H Stamm, Berlin, Germany) and D 050 Instru-Gen (ad-Arztbedarf GmbH, Frechen, Germany), on the elastic modulus (E-Modulus), flexural strength, macro hardness, micro hardness, average roughness, and colour change of the test specimens was investigated. The active components of Impresept are oxalaldehyde and 1,5-pentanedial. These active components are enclosed to the chemical group of aldehydes. Didecyl-dimethylammonium chloride (quaternary ammonium cation) and 1,5-pentanedial are mentioned as active components of Stammopur DR. Sodium perborate and sodium benzoate with the chemical effect of oxidizing connections are the active substances of D 050 Instru-Gen. The used disinfecting agents were prepared with respect to the concentrations and exposure times regarding the manufacturer instructions, for conditioning of the test specimen. Impresept disinfected the test specimen for ten minutes and was used with a concentration of 100%. The Stammopur disinfecting solution was used with an exposure time of 60 minutes and was prepared in a concentration of 3%. The D 050 Instru-Gen disinfecting solution was prepared in a concentration of 2% and the disinfection time of the test specimens was 60 minutes. Dry storage on one hand and distilled water on the other hand were used in addition to the disinfecting agents as control groups. Test specimens, which were used in the distilled water group, were stored for 60 minutes in distilled water. After expiration of the storage period (dry storage control group) and disinfecting period (including the test specimens of the distilled water control group), the test specimens were rinsed with distilled water for one minute and dried manually. Subsequent, the influence on the E-Modulus, flexural strength, macro hardness, micro hardness, average roughness and color change was investigated.

### 2.2. E-Modulus and Flexural Strength

The E-Modulus and the flexural strength were measured using the three-point bending test as per DIN EN ISO 178:2003.

**Figure 2:**
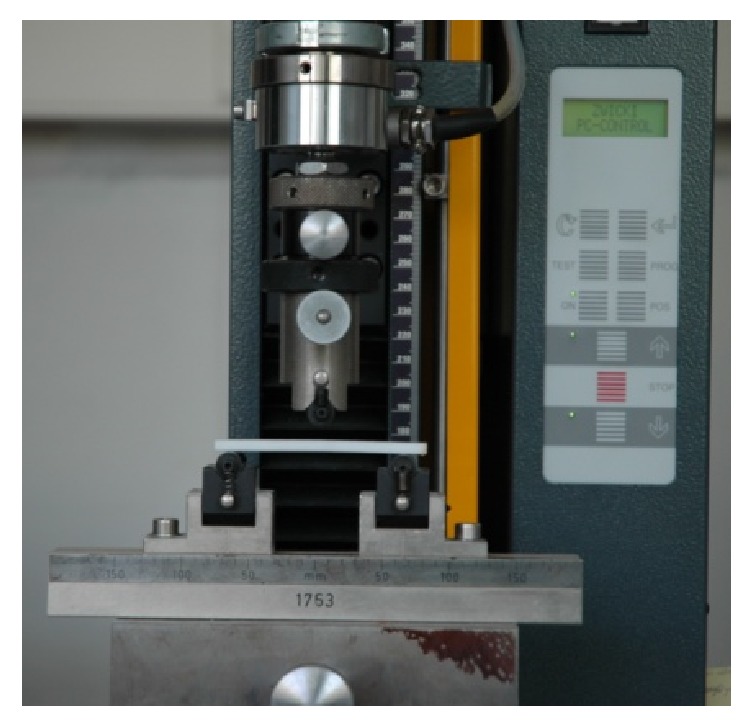
Three-point bending device on the Zwick universal testing machine (ZWICKI TMZW, Zwick GmbH & Co. KG, Ulm, Germany) and Forestacryl colourless test specimen.

120 test specimens were distributed into test groups. The test specimens of the first test group consisted of the stored dry control group. The single storage liquid of the second test group was distilled water. These two test groups were applied as control groups. In the third test group Impresept was used as disinfecting agent. In the fourth test group the influence of Stammopur DR on the test specimens was investigated. The fifth test group was utilized to perform the impact of D 050 Instru-Gen on the test specimens. Following the preparation of each orthodontic acrylic test specimen, three-point bending tests were performed using Zwick machine (ZWICKI TMZW, Zwick GmbH & Co. KG, Ulm, Germany) (Figure 2).

Zwick universal testing machine was constructed performing numerous tests on materials and structures. The investigated test specimens were placed in the universal testing machine between the clamps and the inspection stamp. Each of the tested specimens was mounted on two 5 mm diameter support posts 64 mm apart from each other. This distance was selected to be 16 times the specimens' thickness of 4 mm. A plunger was used to apply a vertical force up to a maximum of 2 kN to the center of the test specimen. The E-Modulus and flexural strength were determined at a cross-head speed of 2 mm/min. The radii of the abutments and plunger were 5 mm. For determination of the E-Modulus and flexural strength, the test speed was kept at a consistent 2 mm/min. Analysis of the resulting data was performed using the test and calibration software testXpert II (Zwick GmbH & Co. KG, Ulm, Germany).

### 2.3. Macro Hardness

**Figure 3:**
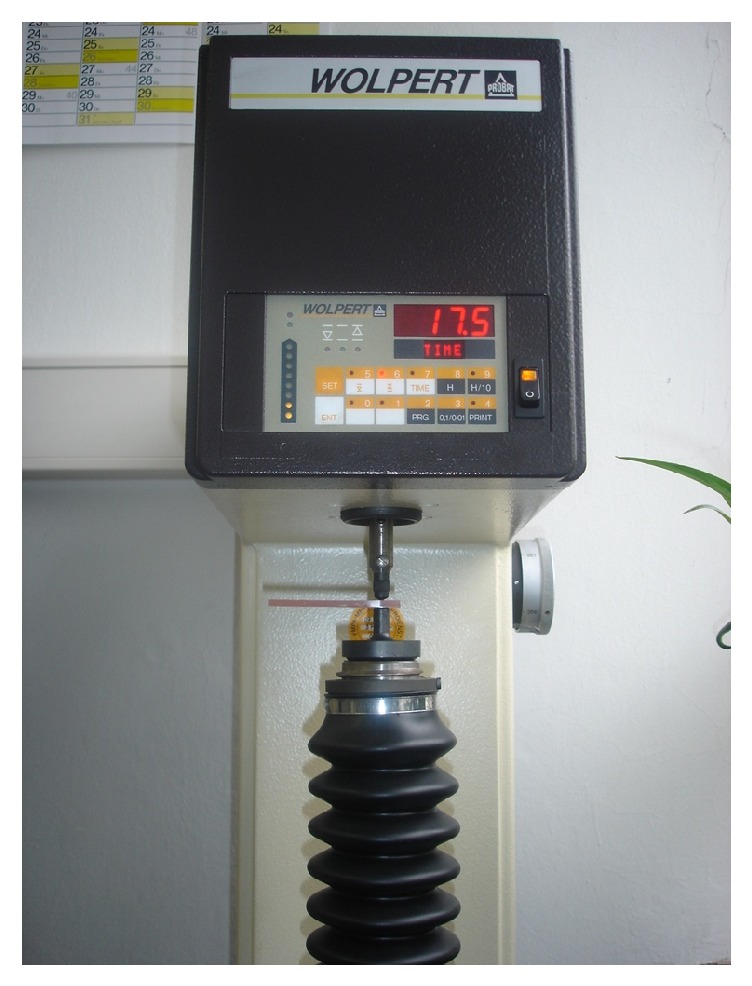
Instron Wolpert- Macro Hardness K-Testors 2524 (Wolpert Wilson Instruments, Pfungstadt, Germany) including a Forestacryl pink test specimen.

The macro hardness of 60 test specimens of the investigated cold-curing orthodontic acrylics was measured by testing the indentation hardness as per DIN EN ISO 2039-1 using the Instron Wolpert-Macro Hardness K-Testors 2524 (Wolpert Wilson Instruments, Pfungstadt, Germany) (Figure 3).

The test load varies between the minimum test force of 49 N and the maximum test force of 961 N unless otherwise stated in the specific testing procedure. The measuring method is according to a measurement under load to evaluate the complete deformation of the investigated orthodontic acrylics. The determined hardness values include elastic, viscoelastic, and plastic deformation components. The distance between each test specimen was 10 mm to avoid the influence of adjacent hardness dots.

### 2.4. Micro Hardness

The micro hardness of 40 test specimens of the investigated cold-curing orthodontic acrylics was measured using a micro hardness test device (Fischerscope H 100C XYp, Helmut Fischer GmbH, Sindelfingen, Germany). The test procedure was performed according to DIN EN ISO 14577-1, 2 and 3. The micro hardness of test specimens was measured in an area of checking which was selected microscopically (Video-Measuring-and Inspection system VMZM-40, 4H-Jena engineering, Jena, Germany). Every single test specimen was charged with a rate of loading of 50mN/s to a maximum strength of 1000mN. Test specimen was discharged after 20 seconds. The depth of impression and the stress of the indenter were registered simultaneously and displayed graphical.

### 2.5. Average Roughness

**Figure 4:**
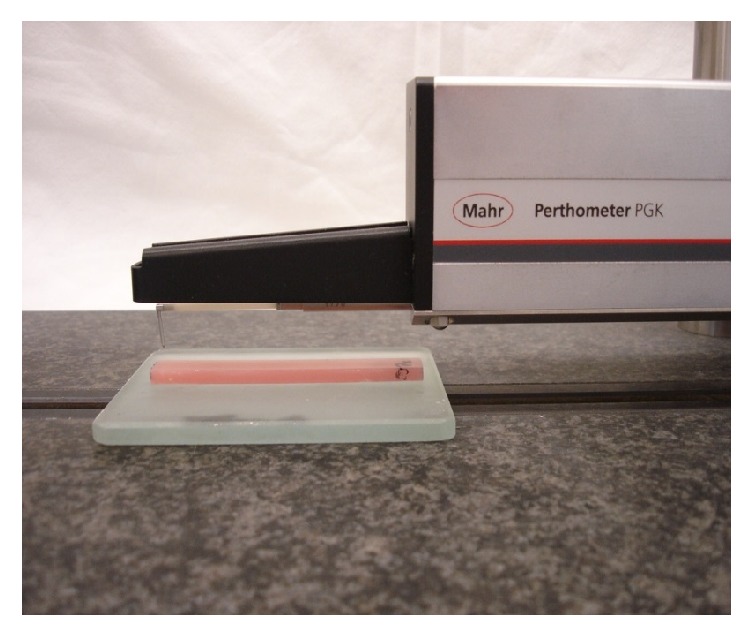
Surface measuring station Perthometer S3P (Perthometer S3P, Mahr GmbH, Göttingen, Germany) measuring a Forestacryl pink test specimen.

For conducting the average roughness investigations, 40 test specimens were used. The average roughness was performed according to DIN EN ISO 4287, 4288, and DIN 4760 using a surface measuring device (Perthometer PGK, Mahr GmbH, Göttingen, Germany) and an evaluation device (Perthometer S3P, Mahr GmbH, Göttingen, Germany) (Figure 4).

The measurements were performed before and after storing in the test liquid. The test track had a distance of 5.6 mm. The entire measuring length was 4.0 mm. The cut-off wavelength *λ*_c_ was 0.8 mm and defined the changeover from surface roughness to ripple.

### 2.6. Colour Change

**Figure 5:**
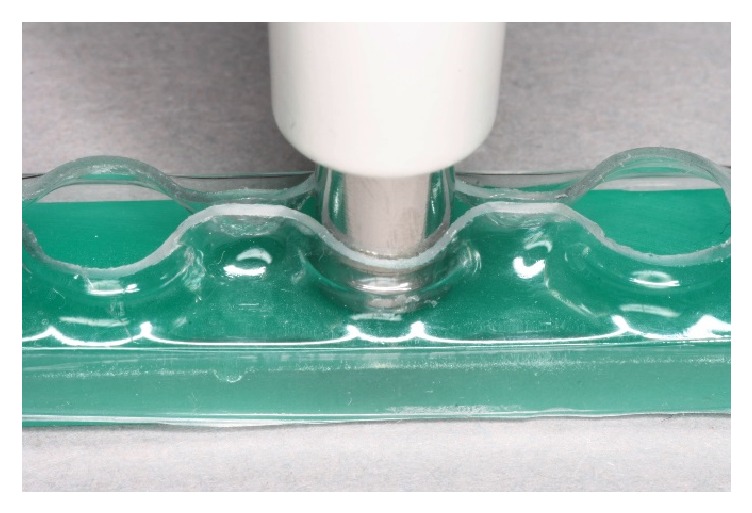
Adapter with sensor tip (Spectrophotometer VITA Easyshade, VITA Zahnfabrik, Bad Säckingen, Germany) measuring an Orthocryl green test specimen.

Colour change of all 260 test specimens of the investigated cold-curing orthodontic acrylics was quantified spectrophotometrically (Spectrophotometer VITA Easyshade, VITA Zahnfabrik, Bad Säckingen, Germany). The test groups were analogue loaded as described above. The spectrophotometer was calibrated prior to collecting colour data from the test specimens. Measurements were performed at ten different positions of each test specimen before and after storing in the test liquid for the defined reaction time of the investigated disinfection agents (Figure 5).

For standardized positioning of the spectrophotometer, a 1.5 mm thick transparent suck-down template was fabricated (Erkodur, Erkodent GmbH, Pfalzgrafenweiler, Germany). A white paper board was used as the background for the specimens during the measurement process to guarantee standardized conditions.

### 2.7. Statistics

Means were calculated, and data were evaluated statistically. Statistical analyses were performed using SPSS 17.0 for Windows (SPSS Inc., Chicago, IL, U.S.A.). Normal distribution of the data was attested (Kolmogorov-Smirnov-test) and significant differences between the groups were detected using the Student's t-test and the single factor variance analysis (ANOVA) was used. The level of significance was set to 5% (p≤0.05). Significant results were analysed using the post-hoc test (Bonferroni).

## 3. Results

### 3.1. E-Modulus

**Figure 6:**
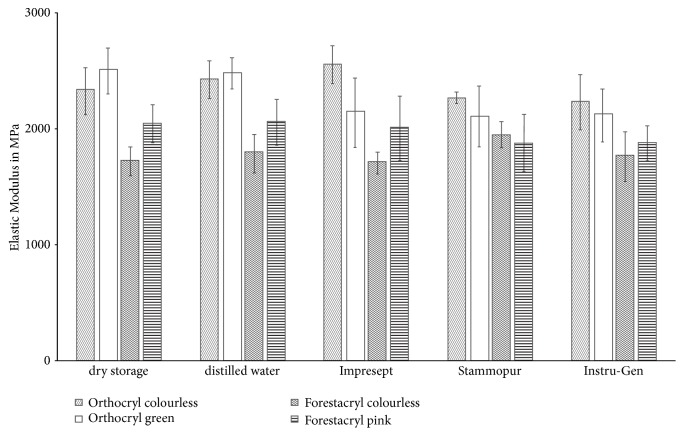
Modification of the elastic modulus of the denture base resins according to the disinfection agents. Results are given as means ± standard deviation.

Results are given as means ± standard deviation (Figure 6). E-modulus data of test specimens which were stored dry ranged from 1720.10 ± 123.20 MPa (Forestacryl colourless – minimum value) to 2494.80 ± 200.60 MPa (Orthocryl green – maximum value). The range of the E-modulus values was from 1783.80 ± 163.80 MPa (Forestacryl colourless – minimum value) to 2474.00 ± 135.00 MPa (Orthocryl green – maximum value) after storage in distilled water. Forestacryl-pink performed a reduction of the E-modulus after disinfection. The slightest change was shown after disinfection with Impresept (2005.17 ± 275.50 MPa) (p>0.05). The maximum variation of the E-modulus was detectable after disinfection with Instru-Gen (1875.0 ± 149.87 MPa) (p>0.05). Changes of the E-modulus after disinfection were found, but not significant in relation to the reference mean values of dry storages and storages in distilled water (Figure 6). This is true for all investigated orthodontic acrylics after disinfection (p>0.05) (Figure 6).

### 3.2. Flexural Strength

**Figure 7:**
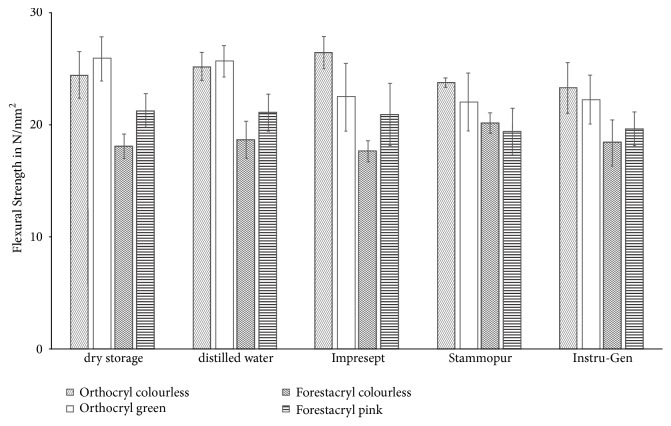
Modification of the flexural strength of the denture base resins according to the disinfection agents. Results are given as means ± standard deviation.

Results are given as means ± standard deviation (Figure 7). For comparable evaluations of the flexural strength, the proof stress limit (*ε*_x_) was set at 1%. Measurements of the flexural strength were taken using all of the different orthodontic acrylics prior to immersion in the tested disinfecting agents and the distilled water control group. Data of flexural strength testing ranged from 18.09 ± 1.07 N/mm^2^ (Forestacryl colourless) to 25.79 ± 1.87 N/mm^2^ (Orthocryl green) of the dry stored test specimens. The range of the flexural strength values was from 18.64 ± 1.59 N/mm^2^ (Forestacryl colourless) to 25.64 ± 1.43 N/mm^2^ (Orthocryl green) after storage in distilled water. The flexural strength of the orthodontic acrylics decreased mainly after disinfection, except for Orthocryl colourless with Impresept (26.37 ± 1.38 N/mm^2^ vs. reference values: dry storage 24.38 ± 2.01 N/mm^2^/storage in distilled water 25.18 ± 1.23 N/mm^2^) and Forestacryl colourless with Stammopur DR (20.14 ± 0.87 N/mm^2^ vs. reference values: dry storage 18.09 ± 1.07 N/mm^2^/ storage in distilled water 25.18 ± 1.23 N/mm^2^) and D 050 Instru-Gen (18.34 ± 2.04 N/mm^2^ vs. reference values: dry storage 18.09 ± 1.07 N/mm^2^/storage in distilled water 25.18 ± 1.23 N/mm^2^) (Figure 7). The disinfection agents had no significant influence on the flexural strength (p≥0.05).

### 3.3. Macro Hardness

**Figure 8:**
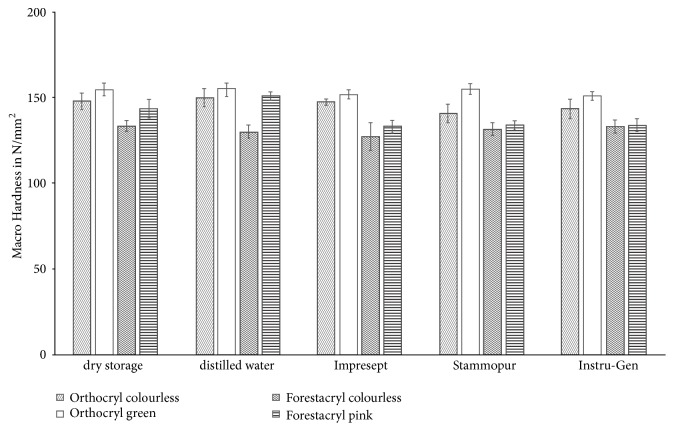
Modification of the macro hardness of the denture base resins according to the disinfection agents. Results are given as means ± standard deviation.

Results are given as means ± standard deviation (Figure 8). Macro hardness values of dry stored test specimens ranged from 132.42 ± 3.94 N/mm^2^ (Forestacryl pink – minimum) to 154.43 ± 3.52 N/mm^2^ (Orthocryl green – maximum) and from 129.69 ± 3.58 N/mm^2^ (Forestacryl colourless – minimum) to 154.42 ± 3.84 N/mm^2^ (Orthocryl green – maximum) after storage in distilled water. In relation to dry storage following significant changes of mean values were shown: Orthocryl colourless performed a significant reduction of the macro hardness after disinfection with Stammopur DR (140.24 ± 5.13 N/mm^2^, p≤0.001) and D050 Instru-Gen (143.13 ± 5.44 N/mm^2^, p≤0.037) (Figure 8). The disinfection of Orthocryl green with D050 Instru-Gen led to significant reduction of the macro hardness (150.63 ± 2.29 N/mm^2^, p≤0.001). This is also true for the disinfection of Forestacryl colourless with Impresept (126.92 ± 7.87 N/mm^2^, p≤0.001) (Figure 8). Changes of the macro hardness values did not show significant results after storage in distilled water.

### 3.4. Micro Hardness

**Figure 9:**
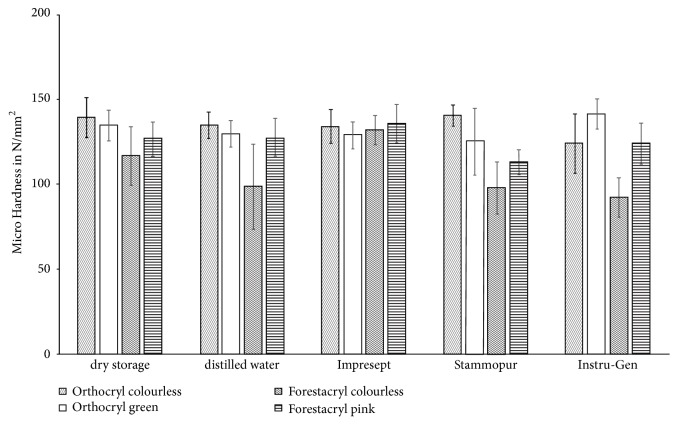
Modification of the micro hardness of the denture base resins according to the disinfection agents. Results are given as means ± standard deviation.

Results are given as means ± standard deviation (Figure 9). Micro hardness values of dry stored test specimens ranged from 116.39 ± 17.05 N/mm^2^ (Forestacryl colourless – minimum) to 139.14 ± 11.55 N/mm^2^ (Orthocryl colourless – maximum) and from 98.43 ± 25.03 N/mm^2^ (Forestacryl colourless – minimum) to 134.78 ± 7.49 N/mm^2^ (Orthocryl colourless – maximum) after storage in distilled water. There was no significant change of the micro hardness detectable after storage in distilled water. Compared to mean values of dry storage following results were found: disinfection of Orthocryl colourless with D050 Instru-Gen led to a significant alteration of the micro hardness (123.87 ± 17.28 N/mm^2^, p≤0.001) (Figure 9). The micro hardness of Forestacryl colourless increased after disinfection with Impresept (131.86 ± 8.52 N/mm^2^, p≤0.039) and decreased after disinfection with Stammopur DR (97.83 ± 15.32 N/mm^2^, p≤0.006) and Instru-Gen (92.14 ± 11.57 N/mm^2^, p≤0.001) (Figure 9).

### 3.5. Average Roughness

**Figure 10:**
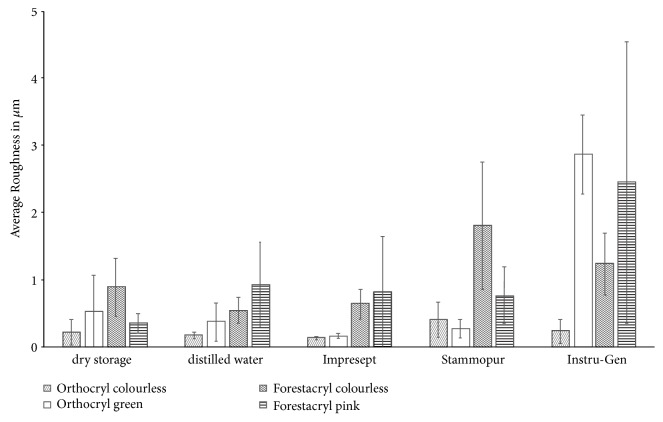
Modification of the average roughness of the denture base resins according to the disinfection agents. Results are given as means ± standard deviation.

Results are given as means ± standard deviation (Figure 10). Average roughness values of dry stored test specimens ranged from 0.22 ± 0.19 *μ*m (Orthocryl colourless – minimum) to 0.89 ± 0.43 *μ*m (Forestacryl colourless – maximum) and from 0.17 ± 0.05 *μ*m (Orthocryl colourless – minimum) to 0.93 ± 0.63 *μ*m (Forestacryl pink – maximum) after storage in distilled water. Compared to the values of the distilled water control group Forestacryl colourless performed a significant increase of the average roughness after disinfection with Stammopur (1.80 ± 0.95 *μ*m, p<0.05). The average roughness of Orthocryl green rose significantly after disinfection with Instru-Gen (2.87 ± 0.59 *μ*m, p<0.05). This is also true for the disinfection of Forestacryl pink with Instru-Gen (2.45 ± 2.09 *μ*m, p<0.05). There was no significant change of the average roughness detectable after disinfecting Orthocryl colourless with any of the investigated disinfecting agent.

### 3.6. Colour Change

**Figure 11:**
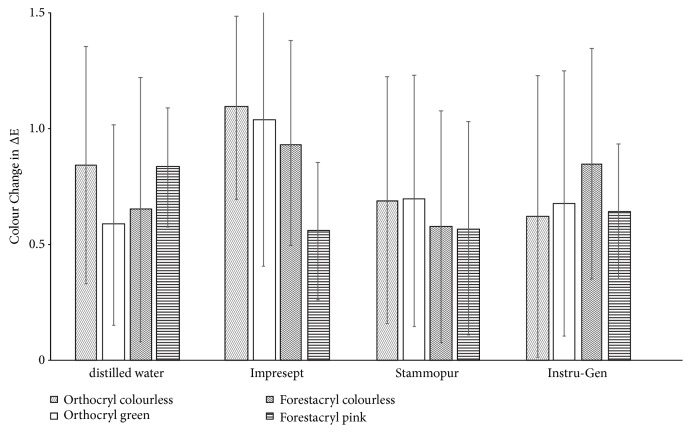
Modification of the colour change of the denture base resins according to the disinfection agents. Results are given as means ± standard deviation.

Results are given as means ± standard deviation (Figure 11). Water immersion of orthodontic acrylics resins in distilled water caused a colour change of the test specimens. ΔE-values ranged from ΔE 0.62 ± 0.25 (Forestacryl pink) in the minimum to ΔE 1.02 ± 0.43 (Orthocryl green) in the maximum after storage in distilled water (Figure 11). The orthodontic acrylics did not perform a significant colour change after disinfection.

## 4. Discussion

In the present* in vitro *study the influence of common dental disinfection agents on elastic modulus, flexural strength, macro hardness, micro hardness, average roughness, and color stability of orthodontic acrylics was determined. Four cold-curing orthodontic acrylics were used [12]. The test specimens were covered by three disinfecting agents containing different primary active ingredients (Impresept, Stammopur DR → aldehydes, D 050 InstruGen → oxidizing connections). Distilled water was used as reference test series to compare the influence of water immersion compared to the possible effect of the disinfecting agents.

Single use disinfection of orthodontic acrylics had no significant effect on elastic modulus and flexural strength (Figures 6 and 7) [13]. The high level of variance of the standard deviation values regarding volume determined elasticity indicates a relatively inferior status of homogenization of the test specimens (Figure 6) [13]. The Orthocryl orthodontic acrylics (colourless and green) performed continuous higher elastic modulus and flexural strength values compared to the results of Forestacryl (colourless and green) (Figures 6 and 7). Additionally, a reduction of the flexural strength after supply of the disinfecting agents and distilled water was not detectable. Therefore, no plasticizing effect could be described. The verification of a plasticizing effect might be possible after a longer storage in the disinfecting agents [14].

Regarding the results of the flexural strength measurements, the outcome of this investigation is hardly comparable to other findings in literature. Therefore, the proof stress limit (*ε*_x_) was set individually at 1%.

In contrast to the results of the elastic modulus and flexural strength, the material-related analysis of the macro hardness, micro hardness, and average roughness of the investigated orthodontic acrylics performed partly significant structural changes after single-shot disinfection (Figures 8, 9, and 10) [15].

However, it has to be considered, if the measured effects on the test specimens are results of the disinfecting process or whether the material related-properties of the cold-curing orthodontic acrylics are responsible for these findings [16]. Therefore, cold-curing orthodontic acrylics were scattered and have pointed out higher inhomogeneity compared to PMMA-based denture base resins [17]. However, another cause may be the manual elaboration and polishing of the used test specimens [18].

All investigated disinfecting agents performed significant changes of the macro hardness on the following orthodontic acrylics: Orthocryl colourless, green and Forestacryl colourless compared to the dry storage control group (Figure 8) [19]. In general, it is recognisable that the Orthocryl acrylics are stronger than the Forestacryl acrylics (Figure 8). Orthocryl green performed overall higher macro hardness results compared to Orthocryl colourless (Figure 8). This finding might be stated, due to the fact that colour pigments are added to the orthodontic acrylics. The other ingredients of the orthodontic acrylics were similar, according to the manufacturer's guidelines. Furthermore, the same polymer powder was used for the scattering process. In this present study, it was shown that the investigated disinfecting agents influenced the orthodontic acrylics after single use disinfection. The measured changes of the macro hardness may clinically lead to a decrease of the overall strength of the used orthodontic acrylics that may lead to an increased risk of fracture of the orthodontic acrylics [20].

The used disinfecting agents performed significant changes of the micro hardness on the orthodontic acrylics Orthocryl colourless and Forestacryl colourless compared to the values of the dry storage control group (Figure 9).

The high standard deviation of the micro hardness results should be critically considered. It is shown that the single values were subjected to strong fluctuations (Figure 9). That may cause in the inhomogeneities of the orthodontic acrylics due to the fact of the described manufacturing process. In this* in vitro* study, test specimens were produced under clinical conditions, using the spreading technique of orthodontic acrylics. Therefore, the manufacturing process of the spreading technique has to be fulfilled exactly. In alternation, polymer and monomer were layered continuously. Neither too much monomer nor polymer must be used and the liquid had to be absorbed completely by the powder [21]. Finally, even by the precise execution of the performed processing method, individual variations of orthodontic acrylics cannot be prevented. Thus, scattered orthodontic acrylics have more structural inequalities than orthodontic or dental base resins which were produced with other processing techniques [22]. Removable orthodontic appliances are mainly used by children and adolescent patients; this could lead to an increased risk of damage by improper handling. Therefore, orthodontic acrylics have to have a sufficient overall strength.

Taken together, the increase of the macro and micro hardness induces an embrittlement of the orthodontic acrylics. This leads to enhanced fracture susceptibility. However, a decrease of the macro and micro hardness stands for a softening of orthodontic acrylics that may cause changes of the shape [19]. In daily clinical practice, considerable disadvantages may arise from the multiple disinfection of orthodontic acrylics.

At the beginning of the measurements of the average roughness, Forestacryl had a higher value (0.89 ± 0.43 *μ*m Forestacryl colourless, dry storage; 0.93 ± 0.63 *μ*m Forestacryl pink, storage in distilled water). In contrast to Forestacryl, Orthocryl showed a reduced average roughness before disinfection (0.22 ± 0.19 *μ*m Orthocryl colourless, dry storage; 0.17 ± 0.05 *μ*m Orthocryl colourless, storage in distilled water) (Figure 10). The disinfection with Stammopur performed a significant increase of the average roughness on Forestacryl colourless (1.80 ± 0.95 *μ*m, p=0.043) (Figure 10). The disinfection with Impresept and D050 Intru-Gen performed no significant influence at the average roughness of any investigated orthodontic acrylic (Figure 10) [23]. However, it should be considered the significantly identified changes of the average roughness possibly caused by the manual processing and the manual polishing of the test specimens. Removable orthodontic appliances are worn by day and at night. During rest phases the removable orthodontic appliances are located in special storage boxes. In that period of time, dental plaque is able to dry up into the surface structure of orthodontic appliances. Therefore, the dental plaque is more difficult to remove. However, the average roughness should not be impaired after the necessary disinfecting process.

The data analysis of the colour measurement shows that the disinfection of the four investigated orthodontic acrylics did not perform any significant colour changes (Figure 11). A minor influence on the colour stability of Orthocryl green was detectable, which was not statistically relevant after disinfection with Impresept and D050 Instru-Gen (Figure 11). Colour change of orthodontic acrylics can be noticed subjectively by human eyes starting from a value of ΔE ≥2. The clinical relevance of these results may be marginal, due to the fact that only ΔE-values higher than 2 are visually perceptible. The ΔE-values of this present* in vitro* study are usually not perceived visually [24, 25].

In summary, some orthodontic acrylics disinfection caused significant changes of the measured parameters. Changes were specific for the applied disinfectant and the tested orthodontic acrylic [11, 13]. Processing of orthodontic acrylics may subject them to numerous possibilities for defects, which result in porosities, shape deviations, and failures of surface structures. Void producing defects impair the structure and downgrade the physical and biological quality of orthodontic acrylics. Additionally, these defects have a negative influence on the hygienic characteristics and they compromise the aesthetics of the orthodontic appliances [16, 25]. Therefore, high pressure is typically used to reduce the described defects during the polymerization process [16, 25, 26].

In principle it is difficult to estimate how often a denture is disinfected during its clinical service time. Dental prostheses have to be disinfected for the first time before delivery [6]. In contrast to dental prostheses, removable orthodontic appliances with a wearing period for at least one year and an inspection interval at a time of six weeks have to be disinfected 20 times or more [27].

Thus, in this study the influence of single use disinfection was tested. In general, disinfecting agents should carry a wide application range. The disinfecting agent Impresept is recommended by the manufacturer for the disinfection of dental impression materials and is certified as surface disinfectant [28]. The universal applicability of disinfecting agents for both dental impressions and orthodontic acrylics is time saving and cost effective. Orthodontic acrylics are one of the most commonly used materials in the orthodontic practice. Thus, it is essential to understand the compatibility of utilized materials and to establish safe as well as standardized hygiene measures in the orthodontic practice [1, 6].

## 5. Conclusions

The objective of all infection control procedures is to prevent transmission of infections between treated patients, orthodontic staff, and orthodontic technicians. Removable orthodontic appliances are subjected to enormous stresses during clinical treatment. Even while integrating and excluding the appliances, additional stress of orthodontic acrylics will proceed. Therefore, it is essential that orthodontic acrylics maintain the elastic modulus and flexural strength after multiple disinfecting processes. In some orthodontic acrylics disinfection caused significant changes of the measured parameters. Changes were specific for the applied disinfectant and the tested acrylic. Thus, from manufacturers of orthodontic resins recommendations for appropriate disinfectants would be desirable.

A limitation of this present* in vitro* study is the investigation of single use disinfection on orthodontic acrylics. This describes newly manufactured removable orthodontic appliances. For simulating the daily clinical practice, further studies should investigate the influence of repeated applications of disinfection agents on orthodontic acrylics. An additional simulation of the clinical situation during the average wearing period of removable orthodontic acrylics could be the investigation of the mechanical properties after disinfection of repaired orthodontic acrylics.

## Data Availability

The data used to support the findings of this study may be released upon application to the Department of Prosthodontics, Martin-Luther-University Halle-Wittenberg, that can be contacted at Dr. Arne Boeckler, Department of Prosthodontics, University Hospital Halle, Magdeburger Straße 12, 06112 Halle, Germany.
